# CISP, an Intrinsically Disordered Cold‐Inducible Barley Protein, Functions as a Small RNA Chaperone

**DOI:** 10.1002/pld3.70179

**Published:** 2026-06-11

**Authors:** Yutaro Okumura, Md. Maksudul Haque, Shin‐ichiro Kidou

**Affiliations:** ^1^ Graduate School of Science Nagoya City University Nagoya Japan; ^2^ Research Center for Biological Diversity Nagoya City University Nagoya Japan

**Keywords:** barley, *CISP*, cold tolerance, intrinsically disordered protein (IDPs), RNA chaperone

## Abstract

Low temperatures are a major environmental stress that limits plant growth and development. Understanding the molecular mechanism of cold tolerance is therefore essential for improving crop performance through molecular breeding. In this study, we focused on CISP, a small, previously uncharacterized protein, specifically induced in barley roots under low temperature conditions, and investigated its potential role in cold tolerance. Heterologous expression of CISP in *Escherichia coli* enhanced late‐phase bacterial growth at low temperatures compared with the wild‐type strain. Similarly, overexpression of CISP in *Arabidopsis thaliana* , which lacks an apparent ortholog, improved seedling growth under low temperature conditions. These results suggest that CISP can promote growth of heterologous organisms used in this study at low temperature environments. To investigate its molecular function, we performed RNA chaperone assays using RNA beacons that tend to form stable secondary structures. CISP reduced the formation of RNA secondary structures and facilitated their destabilization, indicating RNA chaperone–like activity. CISP is a basic, low‐molecular‐weight protein containing an intrinsically disordered region (IDR) at its N‐terminus but lacking canonical RNA‐binding domains such as the cold shock domain (CSD) or RNA recognition motif (RRM). Our findings therefore suggest that CISP may represent a previously uncharacterized type of RNA chaperone. This study provides new insights into the structure and function of CISP and its potential contribution to cold tolerance in barley.

## 1 Introduction

Low temperatures represent a major environmental stress that limits plant growth and productivity. Crops with cold tolerance, such as rice and corn, cannot be efficiently cultivated in high‐latitude or alpine regions (Chinnusamy et al. 2007; Rihan et al. 2017). In contrast, cereals such as wheat and barley exhibit strong cold tolerance, enabling growth in cold climates. Many cold‐tolerant plants have evolved various adaptive mechanisms, including membrane lipid desaturation, accumulation of compatible solutes such as proline, and enhanced activities of antioxidant enzymes such as SOD, APX, and POD (Qian et al. 2024). These mechanisms have been predominantly characterized in aerial tissues directly exposed to cold air. By contrast, the below‐ground root system may rely on distinct and less‐characterized mechanisms to withstand cold stress.

Barley possesses three genes, *CISP1*, *CISP2*, and *CISP3*, which encode small CISP proteins. These genes are strongly induced in barley roots under low temperature conditions (Ying and Kidou 2017). Previous gel shift assays demonstrated that all three proteins can bind single‐stranded RNA (ssRNA) (Ying and Kidou 2017), suggesting a possible involvement of CISPs in root cold tolerance through RNA‐related functions.

RNA molecules are synthesized in the nucleus and serve as templates for protein synthesis in the cytoplasm. However, low temperatures stabilize RNA secondary structures, which impede ribosome scanning and markedly reduce translation efficiency (Rajkowitsch et al. 2007). Impaired translation can lead to metabolic dysfunction and ultimately cell death. To counteract this, organisms produce RNA chaperones—proteins that destabilize excessive RNA secondary structures and maintain translation under stress conditions. For example, the bacterial RNA chaperone CspA from *Escherichia coli* prevents the stabilization of RNA secondary structures at low temperatures and thereby supports translation (Rennella et al. 2017). In *Arabidopsis thaliana* , several RNA chaperones have been identified, including AtCSP3, AtGRP4, and AtGRP7 (Kim et al. 2009; Kwak et al. 2011), and the rice protein WCSP also exhibits RNA‐unwinding activity (Wang et al. 2025).

Most known RNA chaperones contain conserved RNA‐binding domains, such as the cold shock domain (CSD) characterized by a five‐stranded β‐barrel structure, or the RNA recognition motif (RRM), which mediates interactions with nucleic acids (Nakaminami et al. 2006; Ottoz and Berchowitz 2020). These domains are often essential for RNA chaperone function; for instance, the RRMs of AtGRP4 and AtGRP7 are required for their activity (Kwak et al. 2011). RRM‐like domains are also widely conserved in proteins across diverse organisms, including LIN‐28 in *C. elegans* and YB‐1 in vertebrates (Kretov et al. 2019; Moss et al. 1997).

In recent years, intrinsically disordered proteins (IDPs) have attracted attention as key factors in RNA biology. IDPs lack a stable tertiary structure and instead contain intrinsically disordered regions (IDRs) that provide structural flexibility, enabling interactions with diverse partners, including nucleic acids and proteins (Morris et al. 2021). Many RNA‐binding proteins (RBPs) and some RNA chaperones contain IDRs that contribute to their molecular functions. CISP also contains an N‐terminal IDR, but unlike canonical RNA chaperones, it lacks conserved domains such as CSD or RRM.

In this study, we investigated the potential role of CISP in cold tolerance. To assess its biological function, we expressed CISP heterologously in *E. coli* and *A. thaliana* , neither of which possesses an apparent CISP ortholog. We further examined the RNA‐related activity of the protein and evaluated whether CISP exhibits RNA chaperone‐like functions. Through these approaches, we aimed to elucidate the molecular characteristics and functional significance of CISP under low temperature conditions.

## 2 Materials and Methods

### 2.1 Plant Growth Conditions

Barley seeds ( *Hordeum vulgare* L. cv. Minorimugi) were surface sterilized with 2% (w/v) Ca (ClO)_2_ for 30 min, rinsed thoroughly with sterile water, and germinated in the dark at 28°C for 3 days. Germinated seedlings were transferred to a 4°C growth chamber and hydroponically cultivated in 1/5‐strength Hoagland liquid medium. Root and leaf tissues were harvested after 50 days of cold treatment for protein extraction. This prolonged duration was chosen as an experimental requirement to ensure maximal CISP accumulation, based on previous kinetic data showing that *CISP* transcript levels peak at approximately 49 days of continuous cold exposure (Ying and Kidou 2017).

### 2.2 Western Blotting

Root and leaf tissues were separately ground in liquid nitrogen and homogenized in protein extraction buffer (50‐mM Tris–HCl pH 8.0, 100‐mM KCl, 0.5‐mM EDTA, 5% glycerol, 5‐mM DTT, and 1‐mM PMSF). After centrifugation (15,000 rpm, 10 min, 4°C), supernatants were collected, and protein concentrations were determined using the Bradford assay (Bio‐Rad).

A total of 20‐μg protein was mixed with 2× SDS‐PAGE buffer containing 10% 2‐mercaptoethanol and heated at 95°C for 5 min. Samples were resolved on a 15% SDS‐PAGE gel and transferred to a PVDF membrane (0.45 μm; Millipore). Membranes were blocked in 5% (w/v) skim milk/TBST for 1 h at room temperature, incubated with primary antibodies overnight at 4°C, washed, and incubated with secondary antibodies for 1 h at room temperature. Signals were detected using ECL (ImmunoStar, Wako) and imaged with a LuminoGraph II system (WSE‐6200H, ATTO).

### 2.3 Reverse Transcription‐PCR

Total RNA was extracted from 14‐day‐old *A. thaliana* seedlings (WT and CISP1‐overexpressing lines). Tissues were ground in liquid nitrogen and homogenized in RNA extraction buffer (4‐M guanidinium thiocyanate, 0.1‐M Tris–HCl pH 7.5, 1% 2‐mercaptoethanol). Following phenol–chloroform extraction and ethanol precipitation, RNA pellets were dissolved in nuclease‐free water. Samples were treated with RT‐grade DNase (Nippon Gene) and repurified.

First‐strand cDNA was synthesized from 1‐μg total RNA using ReverTra Ace (Toyobo) and oligo (dT) primers. Semiquantitative RT‐PCR was performed using gene‐specific primers for CISP1 (Forward: 5′‐TGGCAATTACCCACACGTCA‐3′; Reverse: 5′‐TGTACAAGCCCTAACACGACAC‐3′) and ACTIN2 (Forward: 5′‐GGTAACATTGTGCTCAGTGGTGG‐3′; Reverse: 5′‐AACGACCTTAATCTTCATGCTGC‐3′) as an internal control. PCR products were separated on 2% agarose gels and visualized under UV light after ethidium bromide staining.

### 2.4 Generation of Transgenic Arabidopsis

The CISP1 open reading frame (ORF) was amplified by PCR and cloned into the pRI101‐AN bar vector, which was transformed into *Agrobacterium tumefaciens* LBA4404. Following cultivation in LB medium, bacterial cells were collected, resuspended in infection medium, and used to transform *A. thaliana* by the floral dip method. CISP1 was introduced into *A. thaliana* ecotype Col‐0 under the control of the CaMV35S promoter.

T_1_ seeds were selected on MS medium supplemented with 100 μg/mL kanamycin. Homozygous T_3_ or later generations were used for analyses.

### 2.5 Phenotypic Analysis of Transgenic Arabidopsis Under Low Temperature

Seeds of *A. thaliana* were surface sterilized in 70% ethanol for 1 min, followed by 1% NaClO for 10 min, and rinsed three times with sterile water. The seeds were plated on MS medium and incubated at 4°C, 15°C, or 23°C. The plates were photographed every 2 days. Leaf area of *Arabidopsis* was quantified using Python/OpenCV‐based image processing with manually optimized HSV thresholding.

### 2.6 Construction and Growth Analysis of Transgenic *E. coli*

The ORFs of CISP1 and CISP2 were amplified by PCR and cloned into the pCold‐1 expression vector (Takara). Constructs were transformed into *E. coli* BL21. Transformed cells were precultured overnight and inoculated into 20‐mL LB medium containing 50 μg/mL ampicillin. After induction with 0.1‐mM IPTG, cultures were incubated at 15°C with shaking. Growth was monitored by measuring OD_600_ at regular intervals.

### 2.7 Protein Expression and Purification

PCR products of CISP1 and CISP2 were cloned into pMD20‐T (Takara) and subsequently excised with *Bam*HI and *Eco*RI for subcloning into pGEX‐6P‐1. Constructs were transformed into *E. coli* BL21. Cultures were grown at 37°C to OD_600_ ≈ 0.6 and induced with 0.1‐mM IPTG for 3 h at 37°C.

Cells were harvested, resuspended in lysis buffer, and lysed by sonication. GST‐tagged proteins were purified using Glutathione Sepharose 4B (Cytiva). GST tags were cleaved with HRV‐3C protease (Novagen). Protein purity and tag removal were confirmed by SDS‐PAGE with Coomassie Brilliant Blue staining.

### 2.8 RNA Chaperone Activity Assay

An RNA beacon mimicking the let‐7 precursor sequence was prepared as previously described (Zlobin et al. 2018) and synthesized by FASMAC. The beacon carried a FAM fluorophore at the 5′ end and a BHQ1 quencher at the 3′ end.

Reactions contained 50‐nM RNA beacon and 5‐μM purified protein (CISP1, CISP2, or GST control) in PBS (20‐μL total volume). To control for potential nonspecific effects arising from the relatively high protein‐to‐RNA ratio (5‐μM protein to 50‐nM RNA) in the assay, purified GST alone was included as a negative control at the same molar concentration (5 μM). This ensured that any observed beacon opening was not an artifact of general macromolecular crowding or high overall protein concentration. Fluorescence was measured every 5 min at the indicated temperatures using a Thermal Cycler Dice Real Time System IV (Takara Bio).

### 2.9 In Silico Analysis

Three‐dimensional structures were predicted using AlphaFold 3 (Abramson et al. 2024). IDRs were predicted using the CAID meta‐predictor (del Conte et al. 2023). A weighted average disorder score was calculated using predictors with confidence scores ≥ 0.5. Protein domain structures were annotated using UniProt (Bateman et al. 2025) and visualized with Illustrator for Biological Sequences (IBS) (Liu et al. 2015).

### 2.10 Statistical Analysis

All quantitative experiments, including *E. coli* growth assays, *Arabidopsis* phenotypic evaluations, and in vitro RNA chaperone activity assays, were performed with at least three independent biological replicates. Data are presented as the mean ± standard deviation (SD) or standard error (SE), as explicitly indicated in the respective figure legends. Statistical significance between the control and target samples was determined using a two‐tailed Welch's *t* test, which does not assume equal variances, to ensure evaluation across diverse stress conditions. A *p* value of < 0.05 was considered statistically significant (*p < 0.05, **p < 0.01, ***p < 0.001). All statistical analyses were performed using Microsoft Excel.

## 3 Results

### 3.1 CISP Accumulates as a Protein in Cold‐Treated Barley

**FIGURE 1:**
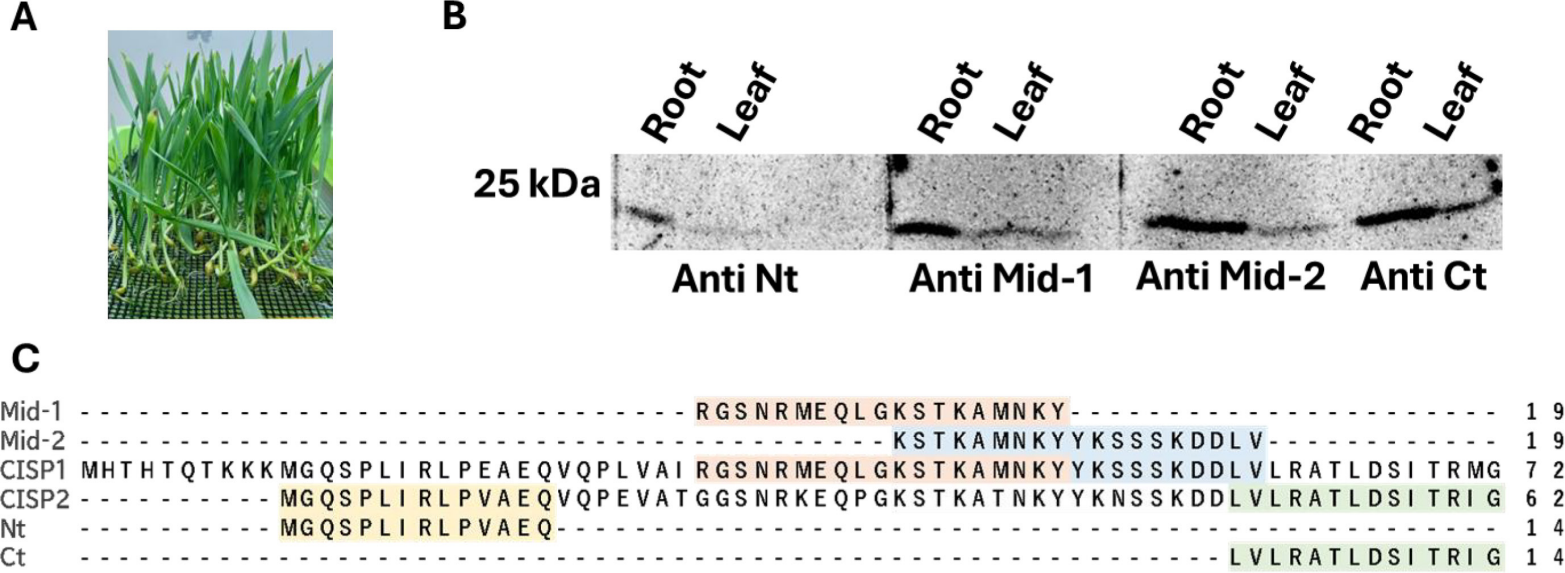
Detection of CISP protein in cold‐treated barley. (A) Fifty‐day cold‐treated barley at 4°C. (B) The results of western blotting. The blots were probed with four independent anti‐CISP primary antibodies, as indicated at the bottom. (C) Schematic diagram of the CISP and amino acid sequences used as antigens for each antibody production.

To determine whether CISP mRNA, which is strongly induced in barley roots under low temperature conditions, is translated into a stable protein, we performed Western blot analysis using protein extracts from cold‐treated barley (Figure 1A). Four anti‐CISP peptide antibodies recognizing distinct CISP amino acid sequences (Figure 1C) were used. All four antibodies detected a band of approximately 25 kDa (Figure 1B). Although this apparent molecular weight is larger than the predicted molecular weight of CISP (∼8 kDa), the fact that all antibodies detected the same band strongly indicates that it represents CISP. Consistent with the mRNA expression pattern, CISP protein accumulation was higher in roots than in leaves (Figure 1B; see also Figure S1 for independent validation in leaves). These results confirm that CISP is synthesized and accumulates in barley roots under low temperature conditions.

### 3.2 Expression of CISP Enhances Cold Tolerance in *E. coli*

**FIGURE 2:**
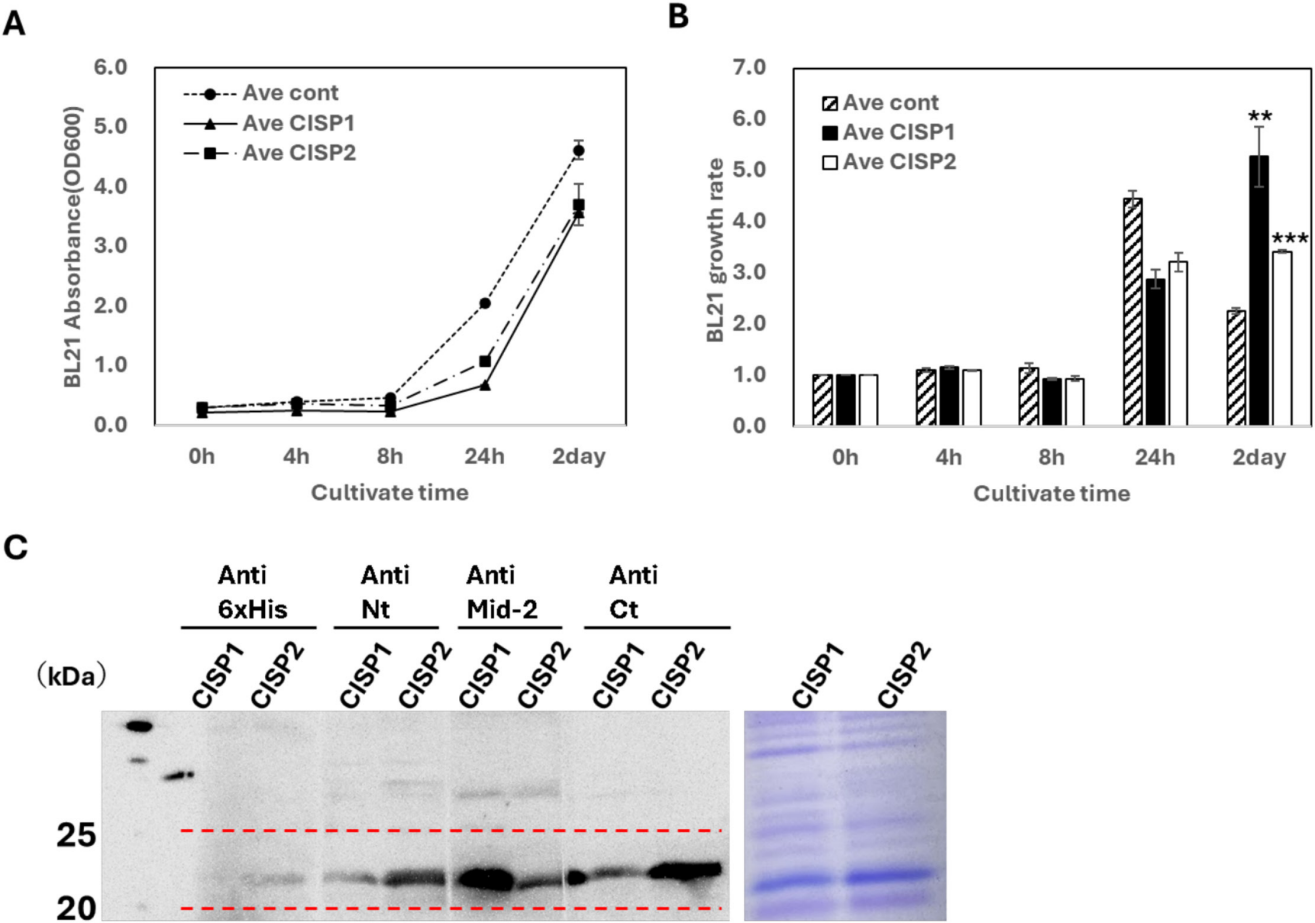
Biphasic growth response of *E. coli* expressing CISP under low temperature. (A) Growth curves of *E. coli* BL21 cells transformed with an empty pCold‐1 vector (Control), pCold‐1‐*CISP1*, or pCold‐1‐*CISP2*. The *E. coli* cells were grown at 15°C after induction with IPTG. Cell growth was monitored by measuring the optical density at 600 nm (OD_600_). Values represent the mean ± standard deviation from three independent experiments (*n* = 3). (B) Specific bacterial growth rates of each strain calculated for distinct cultivation time intervals (0–4 h, 4–8 h, 8–24 h, and 24 h to 2 days) derived from the proliferation data in (A). Panel (B) clearly illustrates the biphasic growth response, characterized by an initial growth suppression during the early phase (0–8 h), followed by a significantly enhanced growth rate in *CISP1*‐ and *CISP2*‐expressing strains during the late phase (24 h to 2 days). (C) Western blot analysis confirming the protein expression of CISP1 and CISP2 in each construct. Total proteins were extracted from induced cells and probed with three types of anti‐CISP antibodies and anti‐6×‐His antibody. Data are presented as mean ± SD (*n* = 3). Asterisks indicate statistically significant differences compared to the control in Panel B (Welch's *t* test: **p < 0.01, ***p < 0.001).

To determine whether CISP confers cold tolerance, CISP1 and CISP2 were expressed in *E. coli* , and their growth was observed over 2 days at low temperature (15°C). *E. coli* growth was measured by determining absorbance at 600 nm using a spectrophotometer. The results (Figure 2A) showed that CISP1 and CISP2‐expressing strains grew more slowly than the control strain ( *E. coli* BL21 transformed with the vector). Therefore, to clearly evaluate the phase‐dependent effects of CISP expression, we calculated the specific growth rates of each *E. coli* strain for the following distinct intervals: 0–4 h, 4–8 h, 8–24 h, and 24 h to 2 days after the start of culture (Figure 2B). We confirmed that the growth efficiency during the late phase, from 24 h (entering the logarithmic growth phase) to 2 days, was significantly higher in the CISP1 and CISP2 expression strains compared to the control strain. Furthermore, Western blot analysis confirmed that either CISP1 or CISP2 was expressed in the two CISP expression strains used for analysis (Figure 2C).

### 3.3 CISP1 Overexpression Enhances Germination and Growth of *A. thaliana* at Low Temperatures

To investigate the *in planta* function of CISP under cold stress, we generated transgenic *A. thaliana* lines overexpressing barley *CISP1* in the wild‐type (WT) Col‐0 background. To strictly evaluate intrinsic cold tolerance and avoid temperature preacclimation, standard seed stratification (dormancy breaking) was intentionally omitted for the initial plate assays.

**FIGURE 3:**
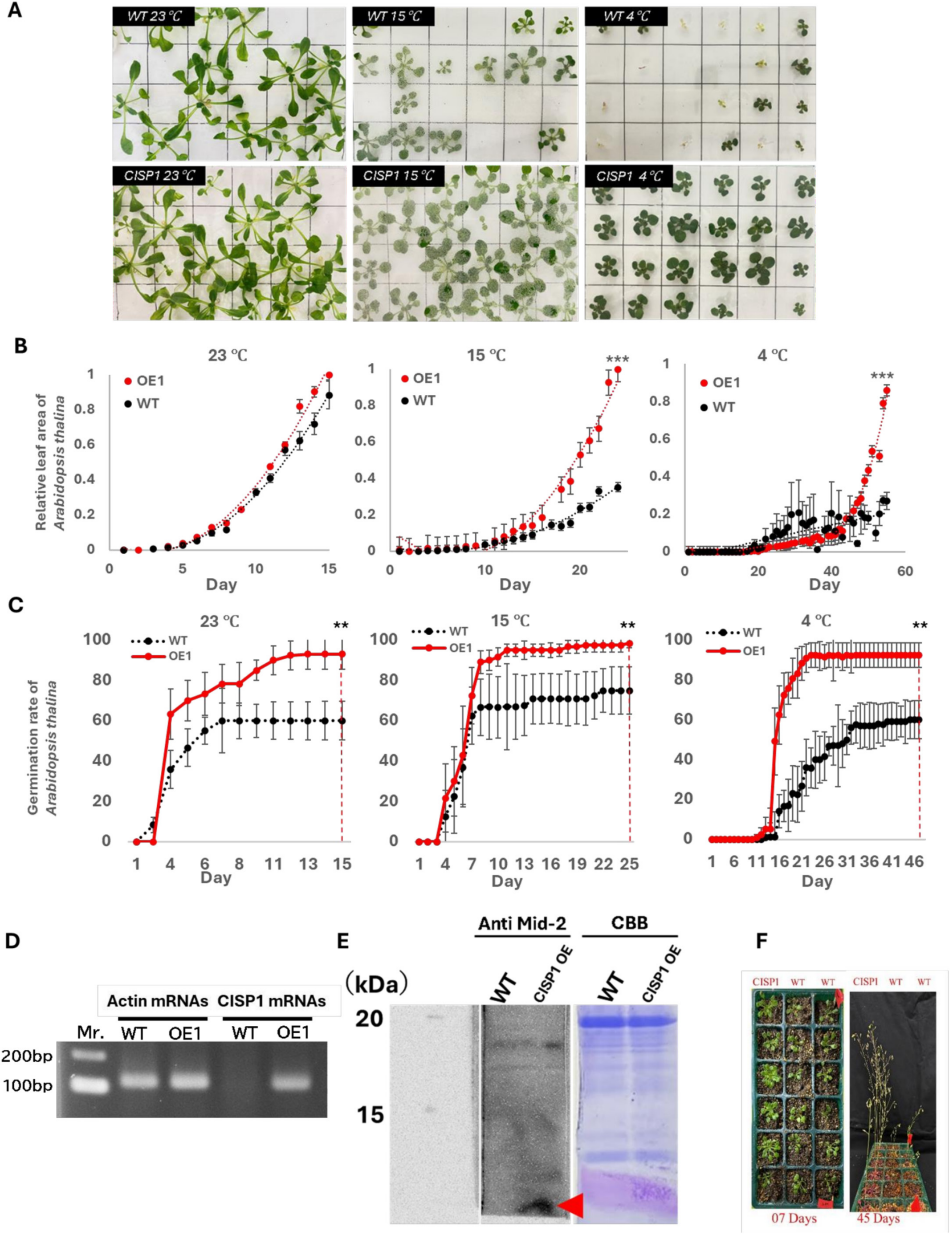
Overexpression of CISP1 improves the cold tolerance of *Arabidopsis thaliana* . (A) Growth levels of 21‐day‐old seedlings of wild‐type (WT [Col‐0]) and CISP1‐overexpressing (CISP1‐OE) transgenic *Arabidopsis* grown at the indicated temperatures (4°C and 15°C). (B) Quantification of rosette leaf area over time. Leaf area was calculated from images of individual plants. Data are shown as average value from 120 plants per line. (C) Germination rate of Experiment A. (D) Expression analysis of the CISP1 gene of wild‐type and CISP1 overexpression plants by RT‐PCR. (E) Western blot analysis confirming CISP1 protein expression in transgenic lines. (F) Growth condition of plants cultivated for an extended period on soil at 4°C. Data are presented as mean ± SE (*n* = 3). Asterisks indicate statistically significant differences compared to the control (Welch's *t* test: **p < 0.01).

We first evaluated the developmental phenotypes of a representative transgenic line (OE1) and WT plants grown on agar plates at 23°C, 15°C, and 4°C (Figure 3A). At the standard temperature of 23°C, no significant differences in relative leaf area were observed between the two lines. However, under constant cold stress at 15°C and 4°C, the OE1 line maintained continuous vegetative growth, resulting in significantly larger relative leaf areas compared to the WT (Figure 3B).

We subsequently quantified the germination rates under these nonstratified conditions. Because dormancy breaking was not performed, WT germination plateaued at approximately 60%. In contrast, OE1 seeds maintained nearly 100% germination across all tested temperatures, including severe cold stress (Figure 3C). The results of the control assays using standard stratified seeds are provided (Figure S2). To confirm that this remarkable cold tolerance is driven by the introduced transgene, the expression of *CISP1* in the evaluated OE1 plants was verified at the molecular level. RT‐PCR analysis detected a distinct *CISP1* transcript band in the OE1 line, which was absent in the WT (Figure 3D). Furthermore, Western blot analysis of total leaf protein revealed a specific signal at approximately 10 kDa in the OE1 extract (Figure 3E). This size closely corresponds to the theoretical molecular weight of the CISP1 monomer (~8.5 kDa), confirming the successful synthesis and accumulation of the CISP1 protein *in planta*.

Finally, to verify that this cold‐tolerant phenotype is reproducible across independent transformation events and natural cultivation environments, a second independent transgenic line (OE2) was grown in soil at 4°C. Consistent with the OE1 plate assays, the independent OE2 plants exhibited sustained development under prolonged cold stress (45 days), whereas the WT plants displayed severe growth inhibition and senescence (Figure 3F). This unequivocally demonstrates that CISP1 effectively confers cold stress tolerance in *Arabidopsis*. Similar morphological growth advantages of the CISP1‐overexpressing line were visually confirmed under moderate cold stress conditions at 10°C and 15°C (Figure S3).

### 3.4 CISP Exhibits Dual RNA Chaperone Activities Under Cold Conditions

**FIGURE 4:**
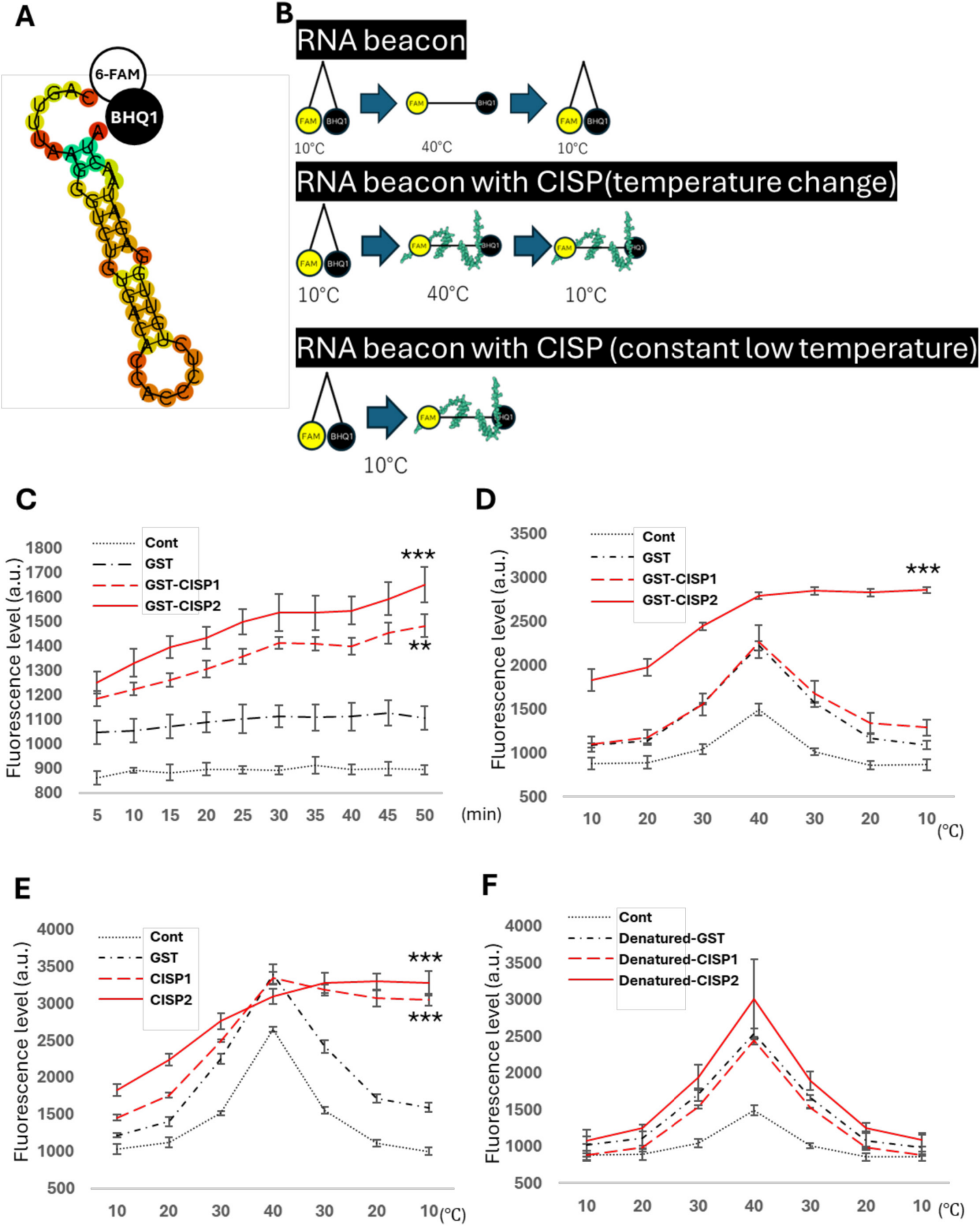
CISP1 and CISP2 possess RNA chaperone activity in vitro. (A) Predicted secondary structure of the let‐7 RNA beacon used in the assay. (B) A model for the RNA chaperone activity of CISP. In the absence of CISP, the RNA beacon reversibly unfolds and refolds with temperature changes. In the presence of CISP, the protein binds to the unfolded RNA and stabilizes its open conformation, preventing it from refolding into the quenched state upon cooling. (C) RNA melting assay. The fluorescence of the RNA beacon was monitored at a constant 10°C in the presence of purified proteins. (D) RNA refolding inhibition assay with GST‐tagged proteins. Fluorescence was measured during a temperature cycle (10°C → 40°C → 10°C). (E) RNA refolding inhibition assay after GST‐tag removal. (F) RNA refolding inhibition assay after heat treatment. CISP1 and CISP2 proteins were boiled at 95°C for 3 min before the assay. Data are presented as mean ± SD (*n* = 3). Asterisks indicate statistically significant differences compared to the control (Welch's *t* test: **p < 0.01).

In this study, RNA melting refers to the active resolution of preformed RNA secondary structures, whereas refolding inhibition describes the prevention of secondary structure re‐formation after denaturation. Importantly, these dynamic chaperone activities are distinct from simple nucleic acid binding, which does not necessarily imply active structural remodeling. Based on prior studies confirming CISP's binding to artificially synthesized single‐stranded RNA, we hypothesized that CISP functions as an RNA chaperone. To test this, we examined the RNA chaperone activity of CISP1 protein synthesized in *E. coli* using a fluorescently labeled let‐7 pre‐microRNA RNA beacon (Figure 4A). RNA beacons have fluorescent dye FAM and the quencher BHQ1 attached to each end. At 10°C, when they form a hairpin structure, they do not fluoresce. However, when the temperature is raised to 40°C, the hydrogen bonds break, the hairpin structure unfolds, and FAM emits fluorescence. Therefore, if CISP possesses RNA chaperone activity that inhibits RNA hairpin formation, fluorescence should persist even when the temperature is lowered from 40°C to 10°C. Conversely, if CISP possesses RNA chaperone activity that resolves RNA hairpin structures, fluorescence should be observed under 10°C conditions (Figure 4B). First, to determine whether CISP could resolve RNA secondary structures, we incubated the RNA beacon, which adopts a hairpin structure, with the protein at low temperature (10°C) and assessed the degree of dissociation. The results showed no change in fluorescence intensity for the control (GST), confirming no dissociation of the RNA beacon. However, in experiments using both CISP1 and CISP2, fluorescence intensity gradually increased over 50 min, confirming that CISP dissociated the hairpin structure of the RNA beacon (Figure 4C).

Next, we evaluated whether CISP could inhibit the reformation of the hairpin structure by the dissociated RNA beacon under low temperature conditions. In a temperature cycling assay (10°C → 40°C → 10°C), the fluorescence of the control sample increased upon heating and decreased upon cooling. This indicates reversible dissociation and refolding of the RNA beacon in response to temperature changes. In contrast, RNA incubated with CISP2 maintained high fluorescence even after cooling, indicating CISP2 inhibits RNA beacon refolding (Figure 4D). However, the GST‐CISP1 fusion protein did not exhibit this activity. Therefore, after removing the GST tag from both CISP1 and CISP2, similar analyses were performed with CISP1 and CISP2 alone. This confirmed that CISP1 also possesses chaperone activity that inhibits RNA beacon refolding (Figure 4E). The reason for this is thought to be that the fused GST tag significantly affected the structure of CISP1, thereby inhibiting its activity. Finally, to confirm that the observed chaperone activity of CISP depends on its structure, we performed the same analysis using thermally denatured CISP protein. The results showed that the ability to inhibit RNA beacon refolding was completely lost (Figure 4F).

### 3.5 CISP Functions as an RNA Chaperone Despite Lacking Canonical RNA‐Binding Domains

**FIGURE 5:**
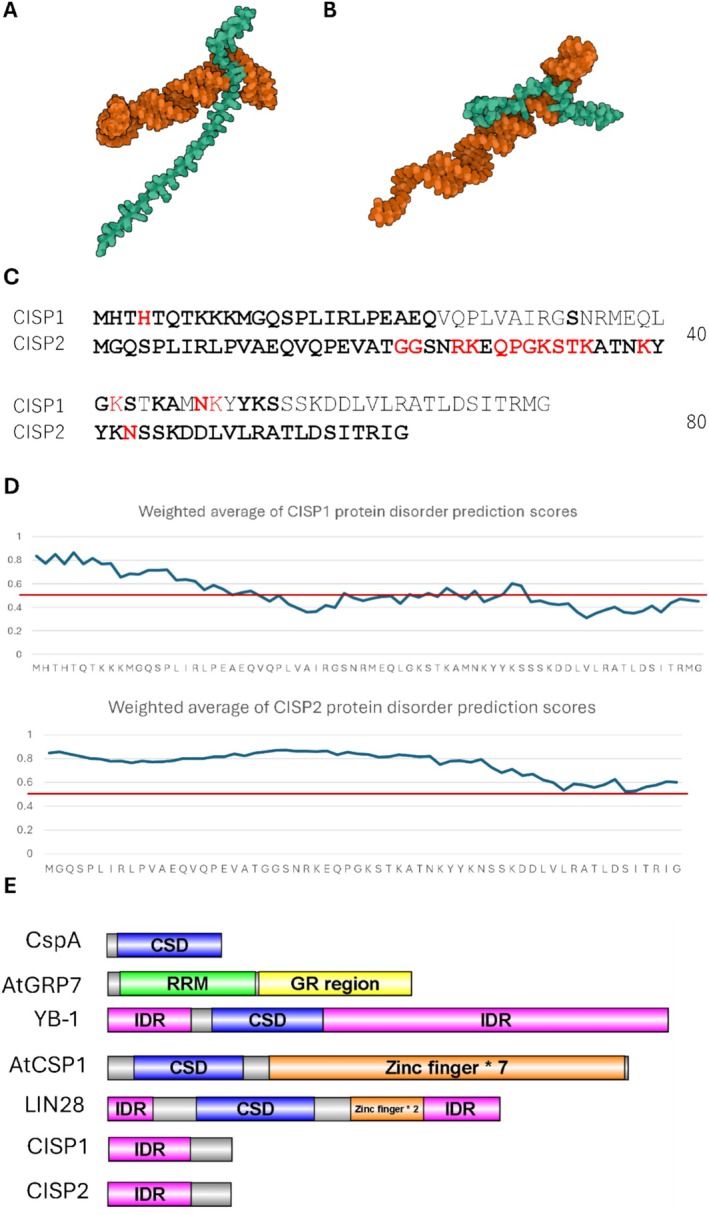
In silico analysis of the RNA beacon and CISP protein features. (A) Predicted three‐dimensional structure of CISP1 in complex with the RNA beacon, modeled using AlphaFold 3. CISP proteins are shown in green, and the RNA is shown in orange. (B) Predicted three‐dimensional structure of CISP2 and RNA beacon, modeled using AlphaFold 3. (C) Amino acid sequences of CISP1 and CISP2. Predicted IDRs (disorder score > 0.5) are shown in bold, and putative RNA‐binding residues are highlighted in red. (D) Prediction of intrinsically disordered regions (IDRs) in CISP1 and CISP2. The *y* axis represents the disorder score calculated by the CAID server; scores > 0.5 indicate a high propensity for disorder. (E) Comparison of domain structures between CISP and canonical RNA chaperone proteins.

It was revealed that CISP1 exhibits chaperone activity toward RNA beacons created with let‐7 pre‐microRNA. Therefore, an in silico analysis was performed on the binding between CISP1 and the RNA beacons. The interaction model between CISP and the RNA beacon was analyzed using AlphaFold 3 (Figure 5A,B). The CISP region indicating RNA binding is rich in basic amino acids (Figure 5C), suggesting that CISP binds to RNA by utilizing its charge. Figure 5D shows the results of examining the disordered regions of CISP1 and CISP2, revealing that both possess IDRs at the N‐terminal end. Figure 5E shows the domain structures of known RNA chaperones. Except for CISP1 and CISP2, these possess CSD or RRM domains. Although CSD and RRM were previously considered crucial for RNA chaperone activity, this study demonstrated that CISP1 and CISP2, lacking these domains, exhibit RNA chaperone activity. This finding provides new insight that small IDP can sometimes confer RNA chaperone activity.

## 4 Discussion

This study advances the analysis of the barley cold‐induced protein CISP. The small size predicted from the CISP gene initially suggested that it might function as a noncoding RNA without translation. However, Western blot analysis confirmed that CISP is indeed translated and functions as a protein. Notably, CISP was detected at a higher apparent molecular weight than predicted, which is likely due to its IDR and highly basic nature, leading to delayed migration in SDS–PAGE. Consistent with this, sequence‐based calculations showed that CISP1 and CISP2 have high isoelectric points (pI) of 9.98 and 9.59, respectively. Such anomalous migration is commonly observed in proteins with high IDR content or high pI (Brocca et al. 2009; Uversky and Dunker 2012). Interestingly, we observed that this electrophoretic behavior is highly context dependent. Although CISP migrates at ~25 kDa in native barley and bacterial systems, it migrates near its true theoretical mass (~10 kDa) in the heterologous dicot *Arabidopsis* matrix. This extreme sensitivity to differing cellular environments or extraction conditions is a classic hallmark of ultrabasic IDPs, further validating its unique biophysical nature.

To investigate the physiological impact of this highly basic IDP under cold stress, we first utilized *E. coli* as a heterologous expression system. Ectopic expression of CISP resulted in biphasic growth under cold stress. Although the precise molecular mechanism underlying this initial delay remains to be fully elucidated, it is possible that the initial growth lag reflects transient, nonspecific electrostatic interactions between the highly basic CISP protein and bacterial nucleic acids during the early stages of cold adaptation. However, under prolonged cold stress, its demonstrated RNA chaperone activity likely assists in maintaining global translation efficiency, thereby significantly improving late‐stage bacterial growth.

Building upon the cold‐tolerance phenotypes observed in bacteria, we next investigated the function of CISP *in planta*. Expressing CISP1 in *A. thaliana* significantly enhanced vegetative growth under severe cold stress (15°C and 4°C). Notably, soil‐grown *CISP1*‐expressing plants successfully reached the reproductive stage under prolonged 4°C exposure, whereas wild‐type plants exhibited severe growth inhibition. These results indicate that CISP1 may function independently in heterologous dicot systems to confer substantial cold tolerance. Although major crops like rice and maize are notoriously cold‐sensitive, our findings suggest that molecular breeding strategies utilizing *CISP1* hold significant potential for improving their cold resilience.

To investigate the molecular mechanism underlying the observed in vivo cold‐tolerance phenotypes, we performed in vitro RNA beacon assays. Recently, the RNA chaperone activity of a homologous protein, BdCISP2, was reported in the model grass *Brachypodium distachyon* (Fadhilah et al. 2026). Building upon this foundational work, our study demonstrates the functional conservation of this mechanism in barley, a major agricultural crop, and expands the conceptual framework by revealing novel biochemical parameters. These assays demonstrated that CISP functions as an RNA chaperone, capable of both resolving pre‐formed RNA secondary structures and inhibiting their initial formation even under sustained, constant low‐temperature conditions. Known RNA chaperones—such as the bacterial CspA family (Jiang et al. 1997), vertebrate YB‐1 (Kretov et al. 2019), and plant WCSP (Karlson et al. 2002; Wang et al. 2025)—typically possess canonical RNA‐binding domains like CSD or RRM (Karlson and Imai 2003). However, CISP lacks these domains. We hypothesize that the highly basic nature of CISP may facilitate electrostatic interactions with negatively charged mRNAs and that its N‐terminal IDR potentially contributes to the structural flexibility required for this chaperone activity. This suggests that well‐defined globular domains may not be necessary for RNA chaperone function, providing insights into alternative structural mechanisms. This critical requirement for unhindered structural flexibility is strongly supported by our technical observations regarding expression vector tags. In the in vivo assays, CISP was functional when expressed with a minimal N‐terminal His‐tag, which is unlikely to cause steric hindrance given the small size of CISP (~8.5 kDa). Conversely, in the in vitro assays using a bulky GST‐fusion construct (~26 kDa), the intact fusion protein exhibited a marked reduction in RNA melting activity prior to tag cleavage. It is probable that the large GST moiety sterically hindered the IDR, restricting its conformational flexibility. Furthermore, the lack of activity in the uncleaved GST‐CISP1 fusion serves as a crucial internal control. It demonstrates that the observed RNA melting is not merely an artifact of simple electrostatic coating by basic residues, as the uncleaved fusion retains the full basic sequence but lacks activity. Therefore, dynamic structural flexibility is a prerequisite for CISP function.

From an evolutionary perspective, clear CISP orthologs are predominantly conserved within Poaceae species, with no apparent orthologs in dicots (Ying and Kidou 2017). This implies that CISP evolved in association with cold adaptation specifically in grasses. However, intrinsically disordered, highly basic RBPs with chaperone‐like activity are ubiquitous across all domains of life (Varadi et al. 2015). Therefore, rather than belonging to a universally conserved sequence family, CISP likely represents a lineage‐specific biological solution utilizing a broadly conserved evolutionary strategy: the deployment of disordered RNA chaperones for cold acclimation.

Future research must focus on elucidating the precise in vivo mechanics of CISP. First, although CISP expression is predominantly pronounced in barley roots, our heterologous assays primarily assessed aerial growth parameters. Detailed root‐specific analyses are necessary to understand the root‐based cold tolerance mechanisms mediated by CISP in its native context. Second, although our findings implicate the N‐terminal IDR in facilitating RNA chaperone activity, empirically determining the precise minimal functional domain remains a critical next step. Future research will require biochemical and *in planta* validation of isolated IDR truncations to fully elucidate this structural requirement. Furthermore, the extended IDR of CISP hints at a potential involvement in liquid–liquid phase separation (LLPS), a mechanism recently highlighted for other IDR‐containing RBPs during stress responses (Xu et al. 2024; Geng et al. 2025). Although LLPS was not directly examined here, exploring this biophysical property, alongside identifying specific in vivo RNA targets using techniques such as RIP‐seq or CLIP‐seq, will be critical next steps. Addressing these questions will deepen our fundamental understanding of how plants leverage IDPs to enact diverse and resilient molecular strategies against environmental extremes.

## Author Contributions

Shin‐ichiro Kidou contributed to the study conception and design. Experimental preparations and data collection were performed by Yutaro Okumura and Md. Maksudul Haque. Data analysis and the first draft were written by Yutaro Okumura and reviewed by Shin‐ichiro Kidou. All authors read and approved the final manuscript.

## Funding

This work was supported by JSPS KAKENHI Grant Number 20K05971 and a research promotion fund (Kyousou Machidukuri) from Nagoya City University, Japan.

## Conflicts of Interest

The authors declare no conflicts of interest.

## Supporting information


**Figure S1:** Validation of cold‐induced CISP protein accumulation in barley leaves.
**Figure S2:** Germination rates of wild‐type (WT) and *CISP1*‐overexpressing (OE1) seeds following standard stratification (dormancy breaking).
**Figure S3:** Morphological phenotypes of wild‐type (WT) and *CISP1*‐overexpressing (OE1) *Arabidopsis* seedlings under moderate cold stress.

## Data Availability

All data generated during the study that are not included in the manuscript are available upon request to the author.
