# Supplementary material for: CISP, an Intrinsically Disordered Cold‐Inducible Barley Protein, Functions as a Small RNA Chaperone
Source: Plant Direct. 2026 Jun 11;10(6):e70179. doi: 10.1002/pld3.70179 (PMC13261088; doi:10.1002/pld3.70179)
Supplement: Supplementary file 1 — Figure S1: Validation of cold‐induced CISP protein accumulation in barley leaves. Figure S2: Germination rates of wild‐type (WT) and CISP1‐overexpressing (OE1) seeds following standard stratification (dormancy breaking). Figure S3: Morphological phenotypes of wild‐type (WT) and CISP1‐overexpressing (OE1) Arabidopsis seedlings under moderate cold stress. [file PLD3-10-e70179-s001.docx]

**Supplementary file**


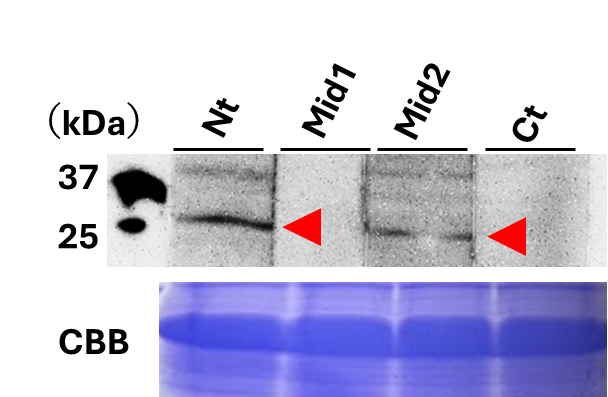


**Supplementary Figure S1. Validation of cold-induced CISP protein accumulation in barley leaves.**

Total protein was extracted from barley leaves grown under normal or cold-stress conditions. To strictly confirm equal protein loading, a parallel gel was stained with Coomassie Brilliant Blue (CBB), with the highly abundant Ribulose-1,5-bisphosphate carboxylase/oxygenase (Rubisco) large subunit serving as a robust loading reference. Western blot analysis using two independent custom anti-CISP antisera (Anti-Nt and Anti-Mid2) reproducibly detected a specific cold-induced band at approximately 25 kDa. The consistent detection of this identically sized band by distinct epitope-specific antibodies unequivocally confirms the detection of native CISP exhibiting anomalous electrophoretic migration.


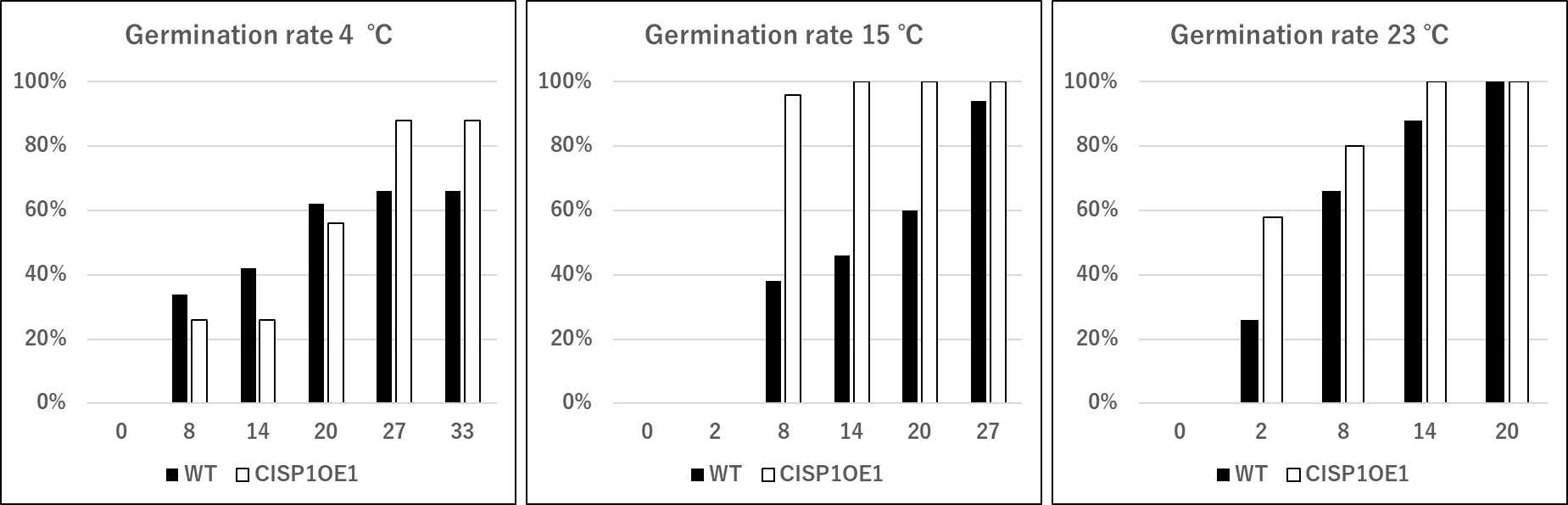


**Supplementary Figure S2. Germination rates of wild-type (WT) and *CISP1*-overexpressing (OE1) seeds following standard stratification (dormancy breaking).**

In an independent experiment from the non-stratified assays (Figure 3C, D), seeds were subjected to a standard cold stratification treatment to break dormancy prior to the germination assay. Under the optimal temperature of 23°C, both WT and OE1 seeds successfully reached 100% germination, confirming that *CISP1* overexpression does not negatively impact baseline seed viability. Under cold stress conditions (15°C and 4°C), WT seeds still exhibited a noticeable delay in germination. In contrast, the OE1 line maintained a rapid germination profile, further supporting the early-stage cold tolerance conferred by CISP1.


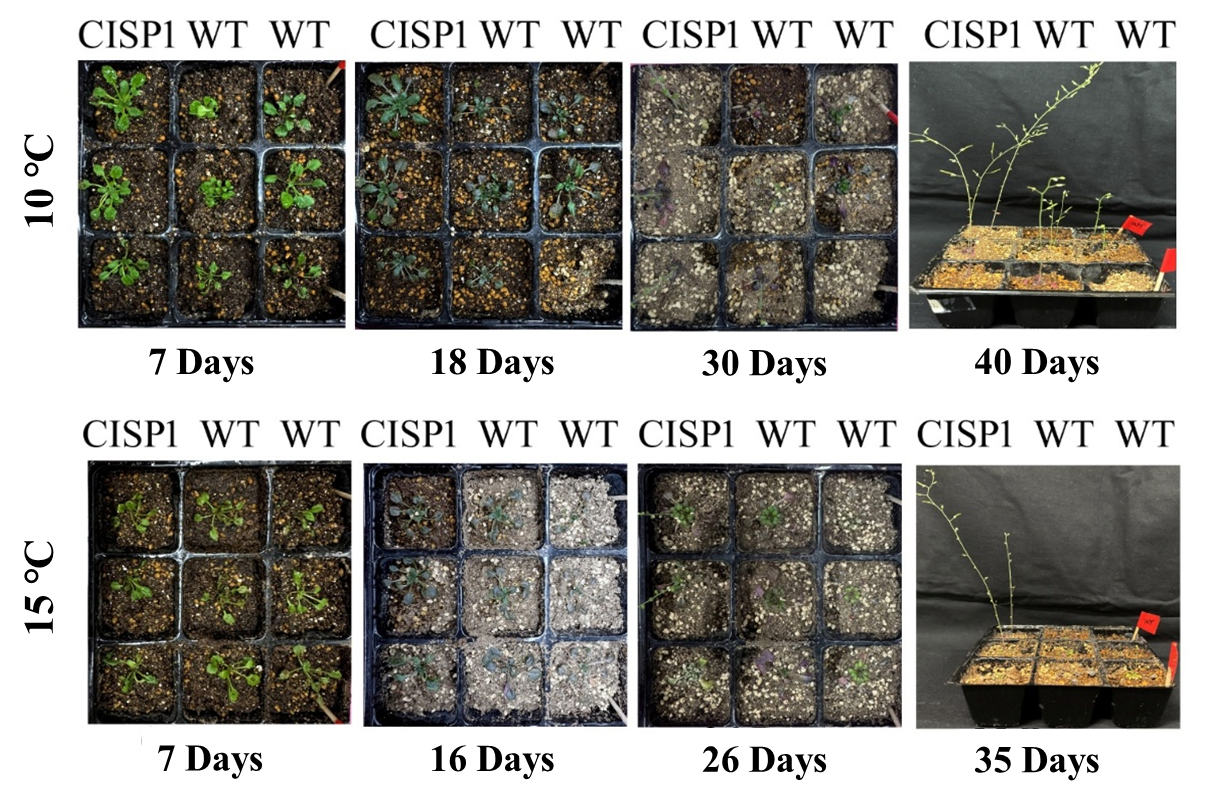


**Supplementary Figure S3. Morphological phenotypes of wild-type (WT) and *CISP1*-overexpressing (OE1) *Arabidopsis* seedlings under moderate cold stress.**

Representative photographs of WT and transgenic OE1 plants grown under constant 15°C and 10°C conditions. Consistent with the quantitative leaf area dynamics presented in Figure 3E, the *CISP1*-overexpressing line exhibits visibly superior vegetative growth, maintaining larger rosette areas and greener leaves compared to the WT under these prolonged cold stress conditions.
